# Host factors influencing the activation of tuberculosis in HIV positive individuals with latent TB infection

**DOI:** 10.1186/1471-2334-14-S3-E16

**Published:** 2014-05-27

**Authors:** Kamakshi Prudhula Devalraju, Sharada Ramseri Sunder, Vijaya Lakshmi Valluri, Arunabala Chowdary

**Affiliations:** 1 LEPRA India – Blue Peter Public Health & Research Centre, Hyderabad, AP, India

## Background

TB kills 1.3 million persons annually, including 275,000 people in India. An estimated 40% of the population in India has LTBI (Latent Tuberculosis Infection), a significant percentage of them being infected with HIV. HIV infection increases the likelihood of LTBI progressing to active TB. Vaccination and immunotherapeutic strategies are alternative approaches that contribute greatly to TB control in HIV+ persons with LTBI.

## Methods

Peripheral blood was drawn from, HIV+ and HIV- individuals with and without LTBI. PBMC and CD14+ monocytes were isolated and 2 million cells were cultured with γ irradiated *M. tuberculosis* H37Rv, CFP-10. Cultures were terminated after 96 hours, and cells were stained for CD4, CD25, FOXP3 and D4GDI. IFN-γ, IL-17, IL-22 were estimated in the supernatants by ELISA. RNA was isolated from CD14 cells and Realtime PCR was performed to quantitate c-maf expression. *P*<0.05 was considered statistically significant.

## Results

In response to *M. tuberculosis*, FoxP3+ cells expand in HIV+ and healthy LTBI+ donors. In contrast, D4GDI+FoxP3+ cells expand only in healthy LTBI+ individuals. IL-17 and IL-22 are significantly high in HIV-LTBI+ individuals, increased c-maf expression in monocytes was observed in HIV+ LTBI+ individuals

## Conclusions

IL-17 and IL-22 can be helpful to control reactivation of tuberculosis in HIV+ individuals.

